# Marangoni-overriding flow in laser-irradiated particle-capped droplets

**DOI:** 10.1073/pnas.2625770123

**Published:** 2026-09-23

**Authors:** Xianglong Pang, Xiaoguang Li

**Affiliations:** ^a^ School of Physical Science and Technology, Shaanxi Basic Discipline (Liquid Physics) Research Center, Northwestern Polytechnical University, Xi’an 710129, China

**Keywords:** marangoni-overriding flow, interfacial jamming, Laplace pressure, nonequilibrium interfaces, laser-driven transport

## Abstract

When a surface tension gradient exists, a Marangoni flow typically occurs from regions of lower to higher surface tension. Here, we report an overriding of this flow in laser-irradiated droplets capped with a jammed particle layer, where particles migrate along the interface toward the apex of reduced surface tension, against the thermal gradient, via a propagating unjamming front. Initial jamming immobilizes light-absorbing particles at the apex, enabling photothermal energy accumulation therein; this removes the central particles and triggers local, intense evaporation that flattens the apex. This flattening steepens the curvature gradient across the unjammed region, amplifying the centripetal Laplace pressure gradient, which dominates over the opposing hydrostatic and Marangoni stresses to drive centripetal transport—a regime we term Marangoni-overriding flow. This flow continually delivers fresh particles to the apex, sustaining the localized energy absorption and thus the intense evaporation that maintains a nonequilibrium curvature field, thereby keeping the Laplace pressure gradient active. This flow regime recasts interfacial jamming as an active control mechanism and, with its tunability via laser power, droplet size, and fluid viscosity, opens avenues for optofluidics, targeted delivery, and nonequilibrium interface engineering.

## Significance

A surface tension gradient across a liquid interface usually drives flow from regions of lower to higher surface tension—a phenomenon known as Marangoni flow. Here, we report a counterintuitive override of this behavior: under laser heating, an initially jammed particle layer accumulates photothermal energy at the apex, driving intense evaporation that flattens the droplet. This amplifies the inward Laplace pressure gradient, overwhelming Marangoni stresses and driving a centripetal flow in the opposite direction. This mechanism redefines interfacial jamming as an active, tunable control strategy rather than a passive barrier, establishing a paradigm for manipulating interfacial transport in soft materials and opening avenues for optofluidics and targeted delivery.

When temperature inhomogeneities or compositional variations generate surface tension gradients at a liquid surface, they induce Marangoni flow—a directed mass transport from regions of lower to higher surface tension. This phenomenon significantly influences fundamental physical processes such as droplet spreading (1), coalescence (2), freezing (3), and bubble migration (4, 5), while enabling controlled material transport with broad application potential in areas including precise particle manipulation (6, 7), enhanced sensing (8), programmable microrobotics (9), and efficient energy harvesting (10). These surface-tension-driven flows also underpin diverse phenomena from coffee-ring pattern tuning (11–14) and Marangoni bursting instabilities (15) to active droplet morphology reshaping (16, 17).

Conventionally, such a flow becomes visually apparent when particle tracers are dispersed on the liquid surface. A necessary, albeit often implicit, prerequisite for Marangoni flow is a fluid interface. The areal particle density must therefore remain below the threshold for interfacial jamming, as jamming effectively solidifies the interface and suppresses tangential flow. Due to its flow barrier properties, interfacial jamming can not only stabilize nonequilibrium shapes (18–22) but also define and stabilize specifically curved interfaces that reach thermodynamic equilibrium (23). Here, we move beyond this static stabilization effect to explore a dynamic regime: when interfacial jamming is locally disrupted, does the restored flow necessarily follow the classical Marangoni direction?

Here we show otherwise: under localized laser irradiation at the center of a particle-capped droplet, a robust centripetal flow emerges, which reverses the direction predicted by conventional Marangoni theory. This reversal is attributed to a self-sustaining feedback loop: localized evaporation flattens the apex, steepening the curvature gradient nearby; the associated Laplace pressure gradient is thereby amplified and drives inward flow; and the flow replenishes particles at the apex, sustaining the evaporation that keeps the loop running. In what follows, we present the core phenomenon and its coupled spatiotemporal scaling, elucidate the apex-flattening mechanism, verify the necessity of interfacial jamming using a high-power, near-jamming control, and finally rule out solutal convection as the primary driver with pure-liquid and microdroplet experiments, supported by a quantitative stress comparison based on lubrication theory.

## Results

**Fig. 1.**
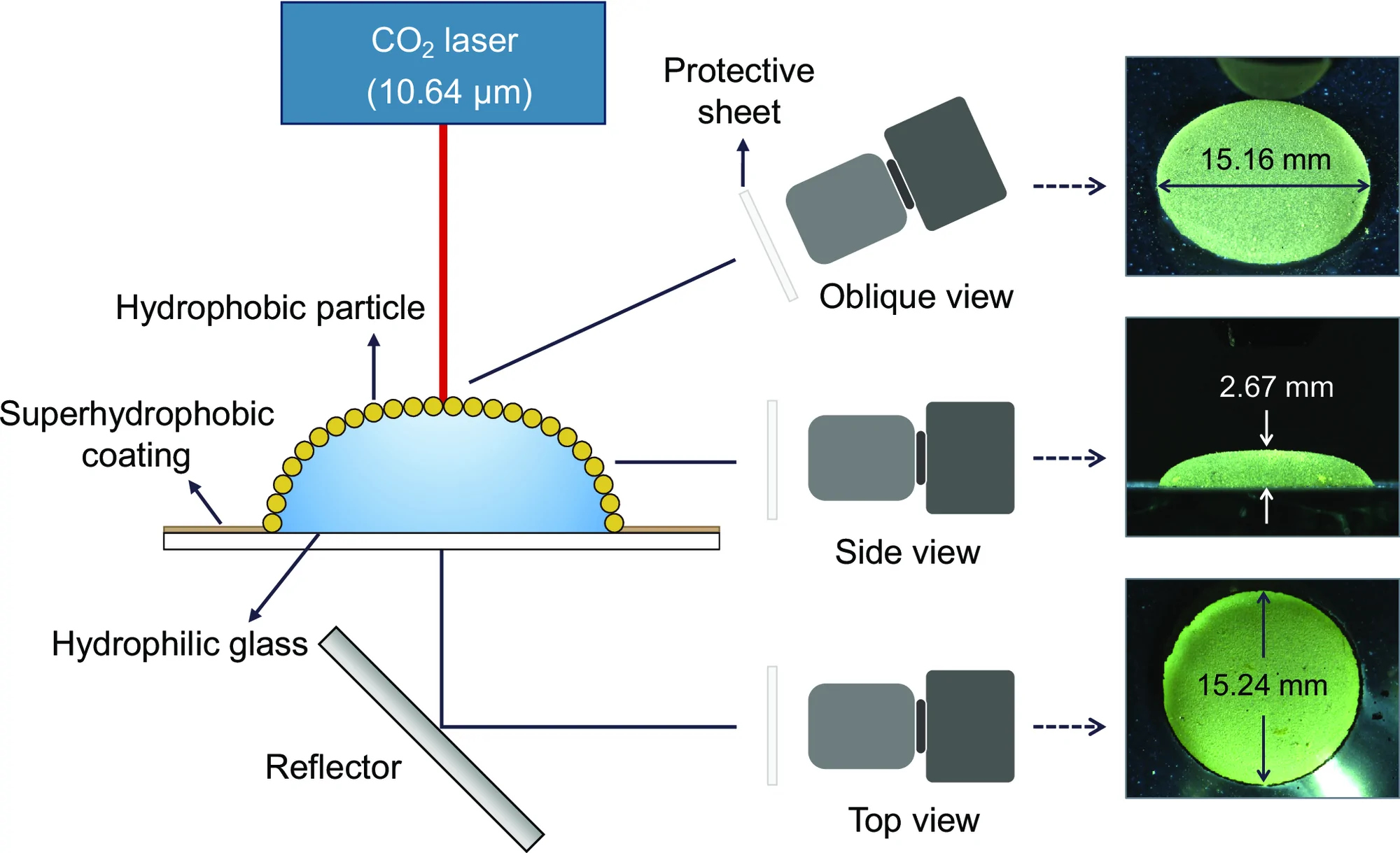
Experimental setup and multiview imaging of the particle-capped droplet. Representative oblique, side, and top views are shown for a 300-μL lycopodium particle-capped sessile droplet containing 70 vol% glycerol–water solution. The droplet is puddle-shaped owing to its large volume and the pinned coalescence formation process. Laser wavelength, 10.64 μm; waist radius, ~0.2 mm; standard power, 8.0 W.

Our core experiments were performed on 300-μL droplets of 70 vol% glycerol–water solution, capped by a critically jammed particle layer. This standard configuration serves as the model system throughout the main text, as it yields well-resolved, highly reproducible interfacial dynamics. Each droplet is confined to a circular hydrophilic region surrounded by superhydrophobic boundaries. The capping layer consists predominantly of a close-packed single layer of hydrophobic lycopodium particles (~45 μm in diameter), with some particle agglomerates distributed across the interface. The layer is formed by coalescing a 100-μL pinned sessile droplet with a 200-μL liquid marble—a spherical droplet fully coated by hydrophobic particles (24, 25). Fabrication details are available in *SI Appendix*, section S1. We focused a 10.64-μm-wavelength, 8.0 W laser at the droplet apex and recorded the resulting interfacial evolution from multiple viewing angles (Fig. 1). Notably, laser irradiation is a common method to induce Marangoni flow by generating absorption-driven thermal gradients (26–30). In our experiment, the laser heats and pyrolyzes particles at the interface center, raising the local temperature and unjamming the irradiated region.

**Fig. 2.**
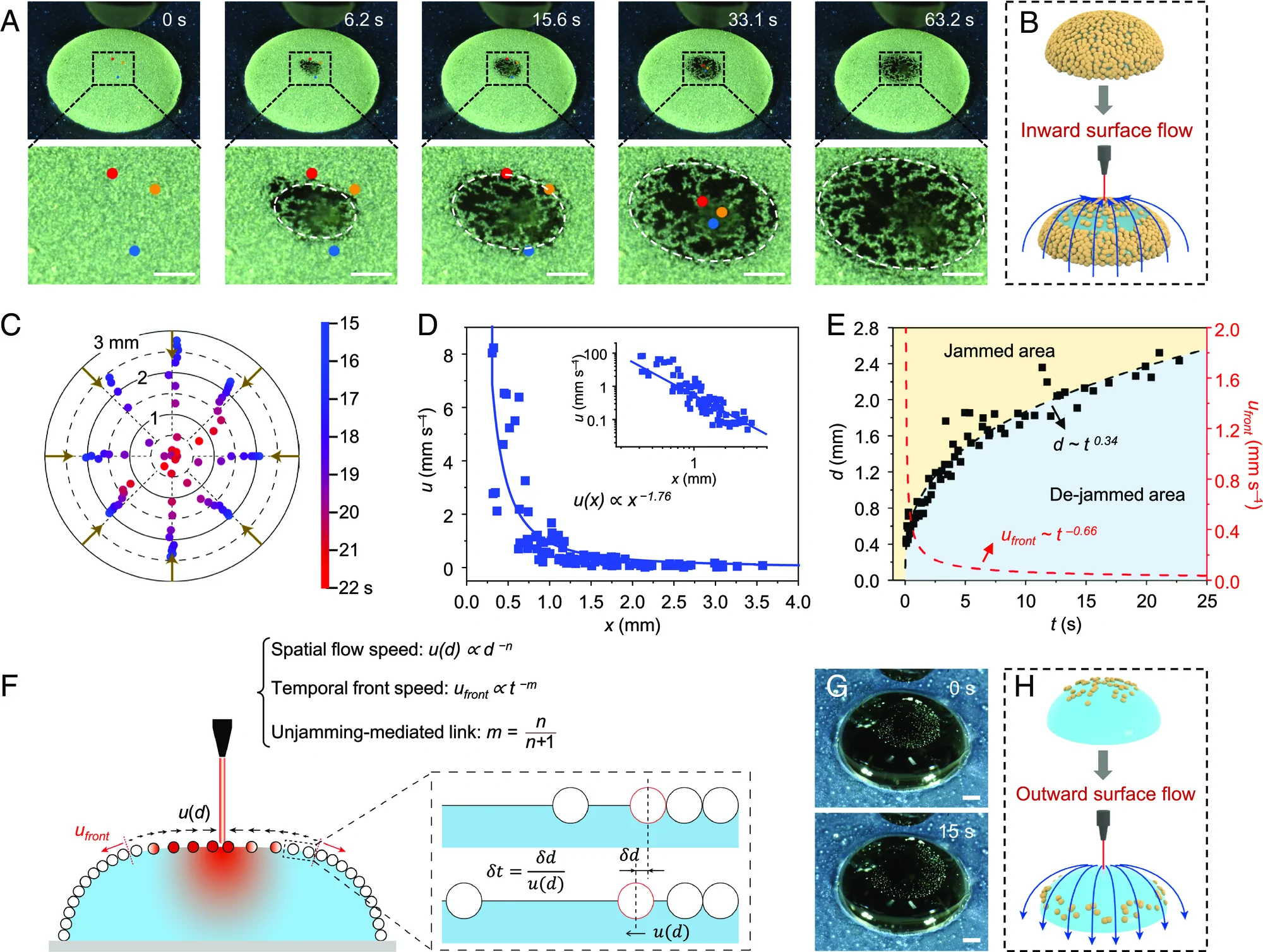
Laser-induced unjamming dynamics and centripetal surface flow. (*A*) Oblique views showing outward expansion of the unjammed region (front marked by white dashed lines). Colored dots mark representative surface regions that remained stationary until the front passed, then moved centripetally. (Scale bar, 1 mm.) (*B*) Schematics of the initially jammed droplet before and during irradiation. (*C*) Centripetal trajectories of eight particle tracers at different azimuths. (*D*) Decay of surface velocity with radial distance from the center. Inset: double-logarithmic plot showing a power-law relationship. (*E*) Temporal evolution of the unjamming front. Notably, radial distances and velocities are measured from top-view projections rather than along the curved interface; this basically does not affect the fitted scaling exponents (*SI Appendix*, section S4). (*F*) Schematic summarizing the coupled spatiotemporal scaling mediated by unjamming kinetics: the spatial power-law decay of surface velocity dictates the temporal decay of front propagation velocity. (*G* and *H*) Images (*G*) and schematics (*H*) of a low-coverage droplet under identical irradiation, showing outward Marangoni flow. (Scale bar, 2 mm.)

Under continuous laser irradiation, an outward-propagating unjamming front expands the mobile, particle-dilute region, while an inward-directed centripetal surface flow transports peripheral particles toward the apex (Fig. 2 *A* and *B*; see also *SI Appendix*, section 2 for macroscopic surface tracer observations). Particles outside the front remain immobilized in the jammed state, and only initiate centripetal migration once the front reaches their position, following a domino-like unlocking sequence. Particles advected to the apex experience a dual fate: continuous white smoke indicates ongoing thermal degradation, while particles are observed moving outward from the center in the droplet interior, confirming their entrainment into a subsurface return flow. Ex situ micrographs (*SI Appendix*, section S3) show that these internalized particles are reduced in size (~30 μm) and darkened, indicating partial pyrolysis. This thermal modification likely renders the originally hydrophobic particles hydrophilic and facilitates their entrainment into the bulk by the descending flow.

To quantify the surface dynamics described above, we begin by tracking particle tracers at multiple azimuthal angles within a fixed radial range of 2.0 to 2.5 mm from the irradiation center to examine the initiation and subsequent progression of centripetal migration (Fig. 2*C*). All particles remain stationary for the first 15.6 s of irradiation, then initiate inward motion synchronously as the unjamming front arrives at this radius, and accelerate steadily as they approach the apex, reaching the center within approximately 6.0 s.

Having established the timing of centripetal migration within a representative radial window, we now examine the spatial structure of the flow across the entire unjammed region. Particle-tracking measurements show that surface velocity *u* follows a power-law decay with horizontal radial distance *x* from the irradiation center: $u\propto x^{-n}$, where the exponent *n* varies systematically with liquid viscosity. For the 70 vol % glycerol–water model system, the exponent *n* ≈ 1.76 (Fig. 2*D*). We next analyze the temporal scaling of the unjamming front. For the same model system, the front position *d* expands subdiffusively with irradiation time *t*, following $d∼t^{0.34}$. Accordingly, the front propagation velocity $u_{\text{front}}$ decays over time as $u_{\text{front}}\propto t^{-0.66}$ (Fig. 2*E*). These two power laws—the spatial decay of surface velocity and the temporal evolution of the front—are quantitatively linked through an unjamming kinetics framework. For jammed particles at the front boundary to escape the static packed state, they must be dragged inward by the local surface flow over a characteristic critical displacement $\delta_{c}$, which breaks interparticle contact constraints and completes the unjamming transition. The characteristic time required for this unlocking step is $\delta t\approx \delta_{c}/u\left(d\right)$, where u(d) is the local centripetal flow velocity at front position d. Since $u\propto x^{-n}$, the velocity at the front follows $u\left(d\right)\propto d^{-n}$. Integrating the characteristic unlocking time from the initial irradiated radius to the current front position yields the total propagation time *t*(*d*), giving $t\propto d^{n+1}$, or equivalently: $d\propto t^{\frac{1}{n+1}}$. Substituting the experimentally measured exponent n≈1.76 for this system yields a theoretical scaling exponent of ~0.36, which agrees closely with the experimental value of 0.34.

The close match above is not coincidental; the agreement improves with increasing viscosity, consistent with the model’s single-particle unlocking assumption (*SI Appendix*, section S4). At a deeper level, this agreement reflects a quantitative scaling mapping between the spatial and temporal velocity profiles: the spatial power law of the centripetal surface velocity, $u\propto x^{-n}$, directly dictates the temporal decay of the front propagation velocity, $u_{\text{front}}\propto t^{-m}$, with the two exponents theoretically satisfying m=n/(n+1). This coupled spatiotemporal picture is schematically summarized in Fig. 2*F*.

Behind these coupled spatiotemporal dynamics lies a driving mechanism that runs counter to conventional Marangoni expectations. Under central laser heating, the radial thermal gradient, combined with preferential water evaporation at the apex in the binary solution, generates a surface tension field that is lower at the center and higher at the periphery. Under such conditions, both thermocapillary and solutocapillary effects should drive outward transport along the interface. This classical outward Marangoni flow is indeed observed under otherwise identical 8.0 W laser irradiation when the initial particle coverage is well below the threshold for interfacial jamming. In this low-coverage control, particles are clearly advected away from the apex (Fig. 2 *G* and *H*; see also Movie S1 for a side-by-side comparison with the standard jammed system in Fig. 2*A*). This stark reversal in flow direction points to an overriding of Marangoni stresses in the initially jammed system. As we demonstrate below, this overriding arises from an amplified Laplace pressure gradient induced by progressive, evaporation-driven flattening of the droplet apex.

**Fig. 3.**
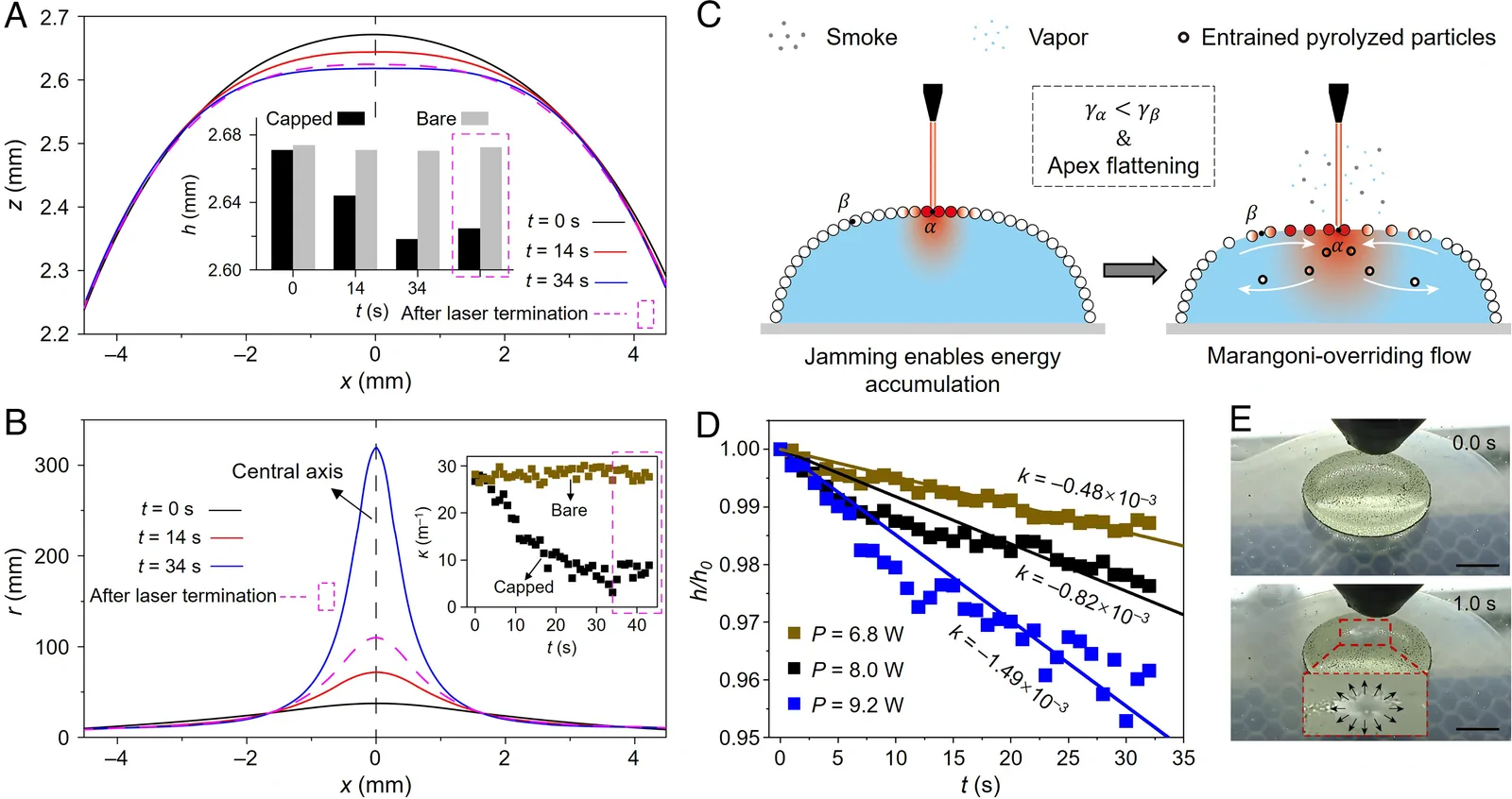
Apex flattening and the Marangoni-overriding flow mechanism. (*A*) Side profiles showing apex flattening over time. Inset: height comparison between capped and bare droplets, confirming that the pronounced flattening arises from the jammed particle layer. (*B*) Radius of curvature *r* with apex curvature *κ* in inset. Dashed lines in (*A* and *B*) indicate recovery after laser termination: the centripetal flow restores the previous curvature, thereby diminishing the Laplace pressure gradient that drives the flow. (*C*) Schematic of the Marangoni-overriding flow mechanism: initial jamming prevents particle advection, enabling energy accumulation and intense evaporation that flattens the apex. This amplifies the centripetal Laplace pressure gradient, driving inward surface flow against the Marangoni direction. (*D*) Affine decay of normalized apex height under three laser powers; *h_0_* is the original height and *k* is the slope. (*E*) High-power control at near-jamming particle coverage under 28.0 W irradiation: particles are dispersed outward (denoted by arrows) by Marangoni stresses, forming a central depletion zone within 1 s. (Scale bar, 5 mm.)

First, we characterize the droplet’s morphological evolution under continuous irradiation using in situ side-profile measurements (Fig. 3*A*; see Methodological Details regarding the rough surface treatment in *SI Appendix*, section 5). The droplet top flattens progressively, a change driven by intense localized evaporation at the laser-irradiated apex. Quantitative curvature analysis reveals a dramatic reshaping of the apex geometry: within 34 s, the apex radius of curvature increases from 37.5 mm to 350.0 mm, corresponding to a drop in curvature *κ* from 26.7 m^–1^ to 3.1 m^–1^ (Fig. 3*B*). This steepens the curvature gradient across the mobile interfacial region near the apex, from the flattened center to the surrounding curved surface, thereby amplifying the centripetal Laplace pressure gradient. Under typical conditions, the Laplace pressure gradient is balanced by the hydrostatic pressure gradient, resulting in a quiescent droplet surface (see mathematical derivation in *SI Appendix*, section 6). In our system, initial jamming prevents light-absorbing particles from being advected away, allowing photothermal energy to accumulate and triggering intense evaporation that flattens the apex, such that the centripetal Laplace pressure gradient is amplified sufficiently to overcome both the opposing hydrostatic gradient and the Marangoni stresses, giving rise to the inward surface flow, a regime we term Marangoni-overriding flow, as illustrated in Fig. 3*C*.

This flow is sustained by a feedback loop: the flow continuously delivers particles to the apex, maintaining the localized energy absorption and thus the intense evaporation that preserves the nonequilibrium curvature field and, with it, the amplified Laplace pressure gradient. Laser-enhanced evaporation is therefore the fundamental driving force, while the Laplace pressure gradient acts as its direct mechanical agent. Conversely, when the laser was terminated, the droplet height and apex curvature partially recovered toward their previous values (dashed lines in Fig. 3 *A* and *B*), and the centripetal flow ceased. Since evaporation is the fundamental driver, controlling its intensity (reflected by the apex flattening) is important. This is readily achieved by adjusting the incident laser power. As the power was increased from 6.8 to 9.2 W, the normalized apex height decay rate increased by a factor of 3.1 (Fig. 3*D*). A quantitative energy-balance model relating laser power to height decay is provided in *SI Appendix*, section S7.

That the apex-flattening rate rises with laser power naturally raises the question of whether sufficiently high laser power can replace the requirement for an initially jammed layer. To test this, we performed experiments at near-jamming particle coverage under otherwise identical conditions, using an elevated laser power of 28.0 W—more than three times that of the standard condition and the highest power safely accessible in our setup. Even under these combined high-power, near-jamming conditions, no centripetal flow was observed: particles in the central region dispersed outward within 1 s (Fig. 3*E* and Movie S2), markedly faster than the low-coverage control in Fig. 2*G*, owing to the stronger capillary gradient generated by the higher input power. This result demonstrates that initial interfacial jamming cannot be compensated for by increasing laser power within the range tested here. Without a preexisting jammed layer to anchor light-absorbing particles at the apex, particles are advected away by Marangoni stresses before sufficient localized energy can accumulate to amplify the curvature gradient.

Beyond the requirement of an interfacial jammed layer, we must rule out alternative driving mechanisms for the observed centripetal flow. Prior studies have demonstrated that solute-driven Rayleigh convection can override Marangoni flow in evaporating binary droplets even at small length scales (31–33). In particular, Diddens et al. offers a rigorous theoretical foundation for this Rayleigh–Marangoni flow competition in binary evaporating systems (33). In our glycerol–water mixtures, preferential water evaporation at the heated apex creates a denser surface layer. On a mobile interface, this heavy fluid flows downward along the profile; it could be hypothesized that in a jammed system it descends vertically, driving bulk recirculation and thus the observed inward surface transport. To test this hypothesis, we performed two complementary control experiments.

**Fig. 4.**
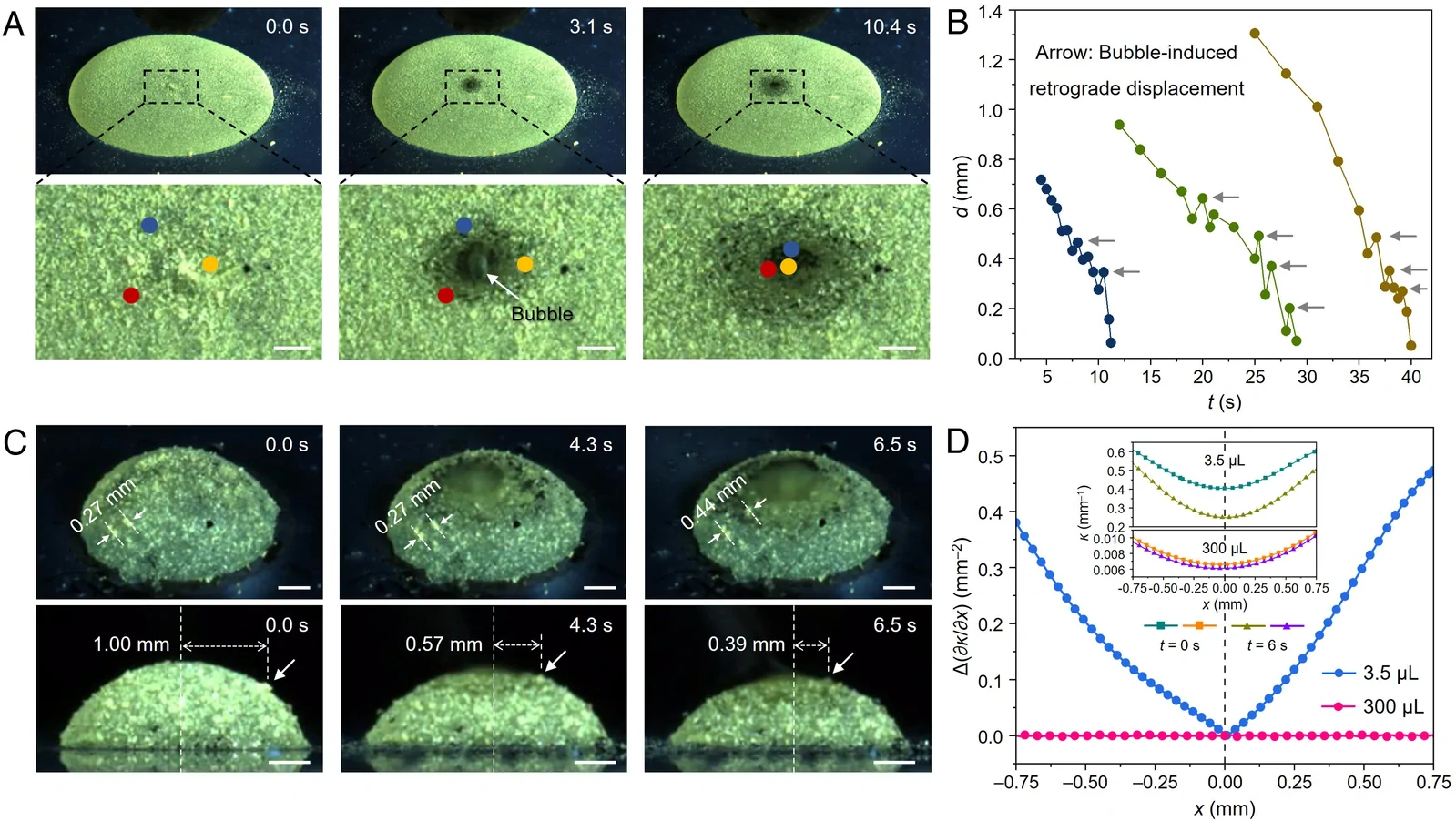
Control experiments using 300-μL pure water and 3.5-μL glycerol–water droplets under 8.0 W laser irradiation. (*A*) Oblique views of a pure water droplet, showing centripetal migration of tracer-marked surface regions (colored dots) and outward expansion of the unjammed area. (Scale bar, 0.5 mm.) (*B*) Distance from the apex versus time for three particle tracers in the pure water droplet, showing an overall decreasing trend with transient retrograde displacements caused by intermittent bubble-induced perturbations. (*C*) Two different 3.5-μL 80 vol% glycerol–water microdroplets during irradiation: *Upper* row, oblique views of one droplet showing the expanding particle-depleted center; the increasing separation between two large particle agglomerates indicates the centripetal flow. *Lower* row, side views of another droplet showing centripetal flow tracked by a large particle agglomerate (arrows). (Scale bar, 0.5 mm.) (*D*) Change in curvature gradient near the apex, Δ(∂*κ*/∂*x*), from 0 s to 6 s for typical 3.5-μL and 300-μL droplets of identical composition (80 vol%). The microdroplet exhibits a larger increase at all positions, consistent with its faster surface flow. *Inset*: raw curvature profiles at 0 s and 6 s for both droplets.

First, we repeated the experiment with 300-μL pure water droplets, where solute gradients cannot exist and all solute-related effects are inherently absent. Time-sequenced images show clear centripetal migration of tracer-marked surface regions, accompanied by the same outward-expanding unjamming front observed in glycerol–water mixtures (Fig. 4*A* and Movie S3). Tracking the distance of individual particle tracers from the apex over time reveals an overall decreasing trend, consistent with sustained centripetal transport; transient retrograde displacements are occasionally observed and attributed to intermittent bubble-induced perturbations near the laser spot (Fig. 4*B*). These bubbles likely arise from dissolved air outgassing rather than bulk boiling: submerged thermocouple measurements showed the liquid temperature beneath the apex remained near 55 °C, well below the boiling point, and their generation was nonvigorous and noncontinuous. This result definitively excludes solutal Rayleigh convection as a necessary condition for the centripetal flow. It is worth mentioning that glycerol–water mixtures, rather than pure water, serve as the primary model system throughout this work: their higher viscosity suppresses bubble nucleation and yields clean, highly reproducible interfacial dynamics, which are essential for the systematic quantitative measurements presented in preceding sections.

Second, we tested 3.5 μL droplets of 80 vol % glycerol–water solution, for which the Archimedes number ($Ar=gh^{3}\rho_{0}\Delta \rho /\mu^{2}$, where μ is the viscosity) is ~0.45, placing the system well below the critical threshold of ~1 for the onset of buoyancy-driven natural convection (32). The centripetal flow not only persists in these microdroplets (Fig. 4*C* and *SI Appendix*, Movie S4), but also exhibits higher surface velocities than the 300-μL system of identical composition when compared at the same radial distance from the apex: at *r* = 0.75 mm, the measured surface velocity reaches 1.7 mm s^–1^ in the 3.5-μL droplet, versus 0.95 mm s^–1^ in the 300-μL system. This trend aligns fully with the Laplace pressure mechanism: smaller droplets have higher intrinsic curvature, so that laser-induced apex flattening amplifies the curvature gradient to a greater degree (compared in Fig. 4*D*) and thus produces a stronger driving force.

Together, these two independent control experiments rule out solutal or buoyant convection as the primary origin of the centripetal flow, providing further support for the evaporation-sustained Laplace pressure mechanism.

**Fig. 5.**
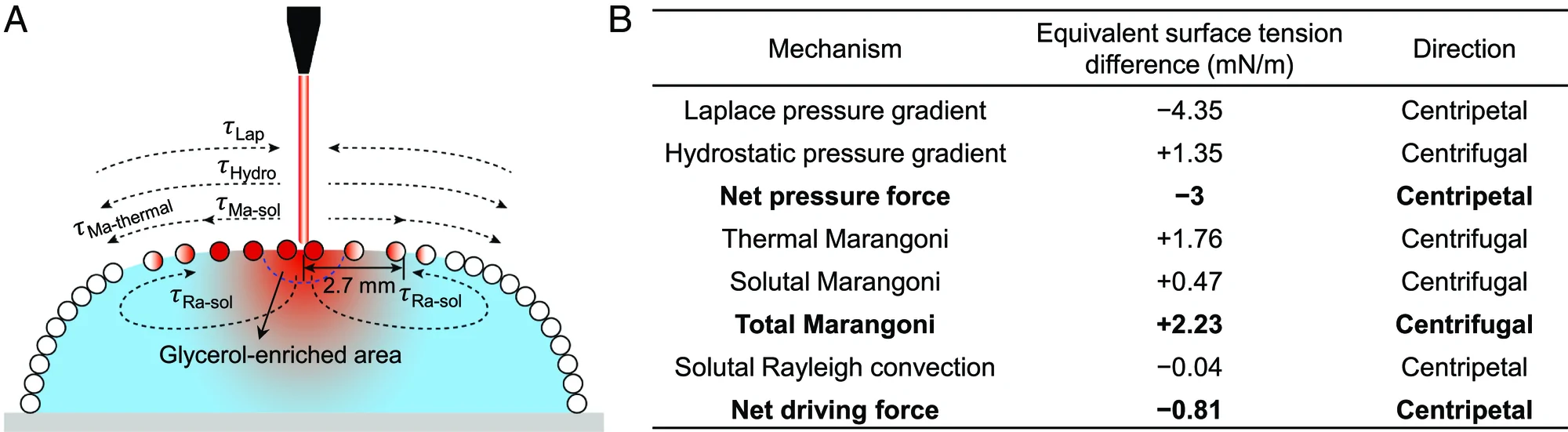
Quantitative stress comparison via lubrication approximation. (*A*) Schematic of the particle-capped droplet (not to scale; see Fig. 1 for the actual profile) under laser irradiation, with the five contributing shear stresses indicated. The analyzed region (0 to 2.7 mm from the apex) remains nearly flat, satisfying the lubrication approximation. (*B*) Comparison of equivalent surface tension differences for the competing mechanisms, showing the dominant inward Laplace contribution relative to the outward Marangoni stresses and the minor buoyancy contribution.

We now return to the 300-μL droplets of 70 vol% glycerol–water solution and place the Laplace pressure mechanism on a quantitative footing by performing an order-of-magnitude stress analysis for the flattened near-apex region via the lubrication approximation, which is valid given the small interface slope and low Reynolds number (~0.14) of the flow. The five contributing shear stresses, including Laplace stress $\tau_{\mathrm{Lap}}$, hydrostatic stress $\tau_{\mathrm{Hydro}}$, thermal Marangoni stress $\tau_{Ma-thermal}$, solutal Marangoni stress $\tau_{Ma-sol}$, and solutal Rayleigh stress $\tau_{Ra-sol}$, are schematically illustrated in Fig. 5*A*.

We start from the standard lubrication momentum equation for creeping flow:

[1] $$\frac{\partial p}{\partial x}=\mu \frac{\partial^{2}u}{\partial z^{2}}.$$

Integrating with a no-slip condition at the substrate (*z* = 0) and a tangential stress balance at the free surface (*z* = *h*) that accounts for Marangoni stress (μ∂u/∂z=∂γ/∂x, where γ is surface tension) yields the surface velocity:

[2] $$u_{s}=-\frac{h^{2}}{2\mu }\frac{\partial p}{\partial x}+\frac{h}{\mu }\frac{\partial \gamma }{\partial x}.$$

To compare driving mechanisms on an equal footing, we define an equivalent surface tension difference Σ such that $u_{s}\equiv \left(h/\mu \right)\Sigma$. The total pressure difference Δp across the comparison interval combines Laplace pressure (γΔκ) and hydrostatic pressure (ρgΔz):

[3] Δp=γΔκ+ρgΔz.

The equivalent pressure-driven surface tension difference is therefore:

[4] $$\Sigma_{p}=-\frac{h}{2}\left(\gamma \Delta \kappa +\rho g\Delta z\right).$$

We perform the calculation for the model system at *t* = 14 s, over the 0 to 2.7 mm radial range of the mobile unjammed zone. Full details of the experimental input parameters, thermocouple-based temperature measurements, and calculation procedures for each stress term are provided in *SI Appendix*, section S8. By convention, a negative Σ denotes centripetal (inward) driving, and a positive Σ denotes centrifugal (outward) driving.

As shown in Fig. 5*b*, the laser-amplified Laplace pressure gradient is the dominant term, with $\Sigma_{\text{Lap}}$ ≈ −4.35 mN/m directed centripetally. The opposing hydrostatic gradient ($\Sigma_{\text{hydro}}$ ≈ +1.35 mN/m) partially offsets this, yielding a net pressure-driven stress of $\Sigma_{p}$ ≈ −3 mN/m. Classical capillary stresses, dominated by thermal Marangoni effects with a minor solutal component, sum to an equivalent outward stress of ~2.23 mN/m. The solutal Rayleigh convection hypothesized above is present in the binary system and acts in the same centripetal direction, but its contribution (~0.04 mN/m) is two orders of magnitude smaller than the net pressure-driven term, serving only as a minor auxiliary effect.

Quantitatively, the net pressure-driven stress alone exceeds the total opposing capillary stresses, confirming that the evaporation-sustained Laplace pressure gradient—after partial counterbalance by the hydrostatic gradient—is sufficient to override classical capillary stresses and sustain the Marangoni-overriding flow. Notably, in pure water, where solute-related effects are absent, the Laplace pressure gradient is the sole driving mechanism and is likewise sufficient to produce the observed centripetal flow.

## Conclusions

In summary, we have uncovered a Marangoni-overriding flow in particle-capped droplets, a transport regime in which the evaporation-sustained Laplace pressure gradient overcomes the combined resistance from the hydrostatic and Marangoni stresses, yielding a net inward driving force. The jammed interfacial layer plays a dual role: it initially confines absorbing particles to trigger intense evaporation and apex flattening, while dynamically releasing a centripetal particle flux along the interface via a propagating unjamming front. This mechanism is quantitatively tunable by laser power, droplet size, and fluid viscosity—parameters that control the driving force, curvature gradient, and flow scaling, respectively. The phenomenon is robust in both capillary-dominated microdroplets and gravity-dominated puddles, and in both pure and binary liquids, indicating that the underlying evaporation-sustained Laplace pressure mechanism operates across droplet size and liquid composition. This establishes a design paradigm for field-responsive soft materials, with broad implications for directed transport, adaptive microfluidics, and the engineering of nonequilibrium interfaces. Extending this mechanism to geometries with nonisotropic curvatures, such as particle-stabilized cylindrical threads where principal curvatures can assume opposite signs, represents a promising direction for further testing and harnessing curvature-gradient-driven flows.

## Materials and Methods

Particle-capped droplets were formed by coalescence of a sessile droplet with a liquid marble on a patterned substrate, yielding a critically jammed monolayer of hydrophobic lycopodium particles (~45 μm). A 10.64-μm laser was focused at the apex, and interfacial dynamics were imaged from multiple viewing angles. Pure-water and 3.5-μL microdroplet controls were conducted under identical irradiation conditions to exclude solutal convection as the primary driver, while high-power near-jamming experiments confirmed the necessity of interfacial jamming. Surface velocities were measured by particle tracking, and temperature was recorded via submerged thermocouples. An unjamming kinetics model was developed to rationalize the spatiotemporal scaling of the surface flow and the unjamming front across different viscosities, and a lubrication-theory-based stress analysis was performed to evaluate the competing driving mechanisms. Complete details of substrate fabrication, droplet preparation, tracer and particle characterization, interfacial profile extraction, and theoretical derivations are provided in *SI Appendix*.

## Supplementary Material

Appendix 01 (PDF)

Movie S1. **Surface flows on initially jammed and low-coverage droplets**. This is a comparative video of laser-induced surface flows. Under laser irradiation, the jammed droplet undergoes continuous unjamming accompanied by centripetal particle migration, a flow direction opposite to classical Marangoni flow; in contrast, the unjammed droplet exhibits outward particle motion consistent with classical Marangoni flow.

Movie S2. **Outward flow on an initially near-jamming droplet under a high laser power**. This video shows phenomena under near-jamming particle coverage and elevated laser power (28.0 W). Particles in the central region are rapidly dispersed outward within 1 s by strong Marangoni stresses, then accumulate and jam at the periphery of the depleted zone, demonstrating that this high laser power does not substitute for a pre-existing jammed layer.

Movie S3. **Centripetal flow on an initially jammed pure water droplet**. A 300-μL pure water droplet exhibits the same centripetal migration and outward-expanding unjamming front observed in glycerol–water mixtures. Intermittent bubbles near the laser spot cause transient outward deflection of nearby particles, but the core centripetal flow pattern remains unambiguous. This result excludes solutal Rayleigh convection as the necessary driver.

Movie S4. **Centripetal flow on initially jammed glycerol–water microdroplets**. This video shows two 3.5-μL microdroplets containing 80 vol% glycerol–water solution, displayed simultaneously in side view (left) and oblique view (right). Both microdroplets exhibit fast centripetal flow, consistent with the Laplace pressure mechanism.

## Data Availability

All Study data are included in the article and/or supporting information.
