# Supplementary material for: Marangoni-overriding flow in laser-irradiated particle-capped droplets
Source: Proc Natl Acad Sci U S A. 2026 Sep 23;123(39):e2625770123. doi: 10.1073/pnas.2625770123 (PMC13624651; doi:10.1073/pnas.2625770123)
Supplement: Supplementary file 1 [file pnas.2625770123.sapp.pdf]

Supporting Information for
Marangoni-overriding flow in laser-irradiated particle-capped droplets

Xianglong Pang & Xiaoguang Li*

*Xiaoguang Li
Email: lixiaoguang@nwpu.edu.cn

This PDF file includes:

Supporting text and Figures (Section 1-8)
Legends for Movies S1-S4
SI References

Other supporting materials for this manuscript include the following:

Movies S1-S4

Section S1: Preparation of particle-capped droplets with interfacial jamming

Patterned substrate fabrication. As illustrated in Fig. S1a, patterned substrates were fabricated on glass slides via a tape-assisted patterning process. A black adhesive tape was applied to the glass surface, and a circular pattern (15 mm diameter, matching the droplet contact diameter) was inscribed using a UV laser. The tape outside the circular region was removed to expose the glass for superhydrophobic coating deposition. Two superhydrophobic coating formulations were used across different experimental batches, with comparable wetting properties and no measurable influence on droplet flow behavior. The alkylated SiO_2 collosol-derived sol–gel coating described in our previous work (1) was adopted for the majority of experiments; for supplementary measurements, an alternative coating was prepared by casting a 3 wt% ethanol dispersion of hydrophobic SiO_2 nanoparticles onto the exposed substrate surface. After coating deposition, the tape covering the central circular region was peeled off, resulting in a hydrophilic central area surrounded by a superhydrophobic border.

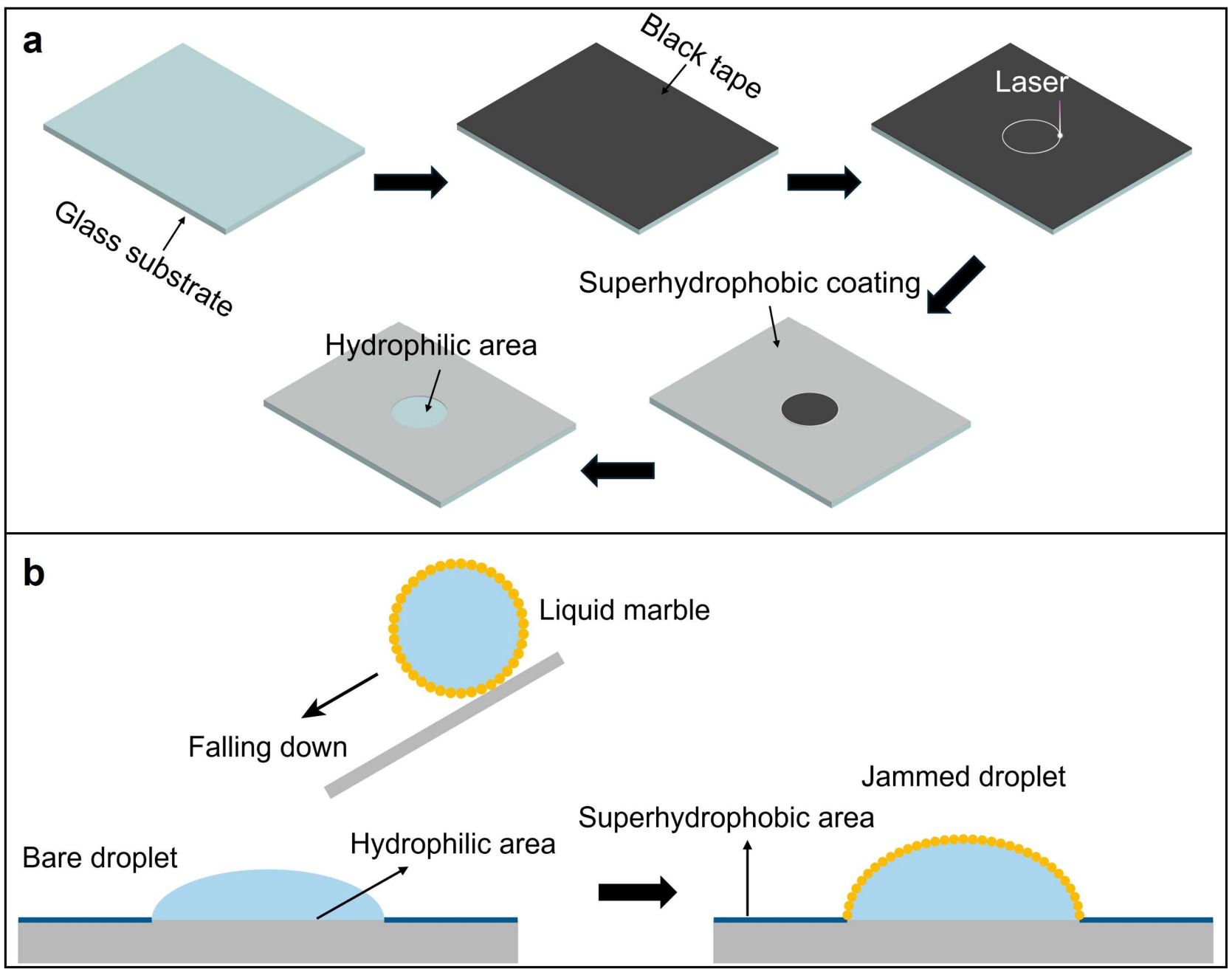

Fig. S1. a) Fabrication process of the patterned hydrophilic–hydrophobic substrate; b) Formation of the particle-capped droplet with a critically jammed interface.

Droplet preparation. As shown in Fig. S1b, a 100-μL bare droplet is first deposited onto the hydrophilic region of the patterned substrate. Subsequently, a 200-μL liquid marble coated with lycopodium powder particles (prepared via rolling a droplet on the powder) is brought into contact with the sessile droplet from above, leading to coalescence. Finally, the substrate is gently shaken to ensure uniform distribution of the particles over the entire droplet surface. In this manner, the

resulting droplet becomes fully capped by lycopodium particles, achieving a critically jammed state in which the surface particles are immobilized while the droplet maintains a liquid-like profile. Notably, substrate patterning is utilized for two reasons: 1) ensuring a circular footprint; 2) inhibiting droplet spreading during coalescence and shaking—thereby ensuring the formation of interfacial jamming.

Section S2: Observation of a macroscopic surface tracer during laser irradiation

To further visualize the direction of surface flow and particle transport, a circular submillimeter plastic piece was used as a macroscopic tracer and placed on the surface of the particle-capped droplet. As shown in Fig. S2, the tracer remained stationary until the unjamming front reached its position. Upon arrival of the front at t = 16.0 s, the tracer began moving centripetally. Notably, the tracer's motion speed was lower than that of the surface flow, attributable to its large size and weight.

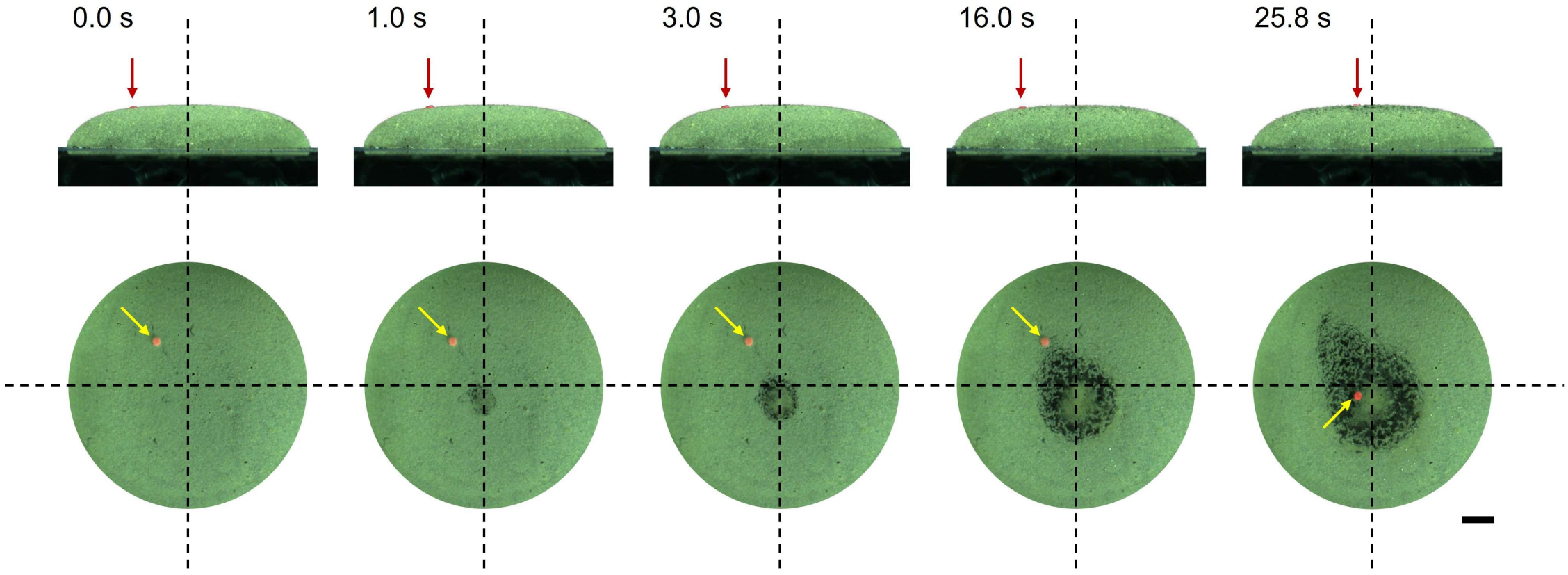

Fig. S2. Side and top views (upper and lower rows, respectively) of a particle-capped droplet marked with a tracer (indicated by arrows) during laser irradiation, illustrating the direction of surface flow and particle transport. Notably, the apparent increase in particle density at the center observed in the top views—compared to the oblique views presented in the main article—originates from particles immersed during laser irradiation, which are captured along the observation path (from bottom to top). Scale bar: 2 mm.

Section S3: Microscopic characterization of internalized particles after laser irradiation

After a predefined period of laser irradiation, the droplet along with its patterned substrate was transferred to an optical microscope for ex situ morphological characterization. The particle-depleted unjammed region at the droplet apex enables sufficient light transmission to visualize particles at different depths inside the droplet.

Images were captured at two focal planes: the droplet apex surface (z = 0) and a plane 1.46 mm beneath the apex (z = –1.46 mm). As shown in Fig. S3, particles entrained into the droplet interior are smaller in diameter than the intact lycopodium particles remaining on the surface. The

internalized particles also appear darkened, in contrast to the pale-yellow color of unmodified surface particles.

These changes in particle size and color indicate partial thermal pyrolysis of the lycopodium spores induced by laser heating at the apex. This thermal modification is expected to alter the surface chemistry of the originally hydrophobic particles, increasing their hydrophilicity and promoting their detachment from the liquid surface and entrainment into the subsurface return flow.

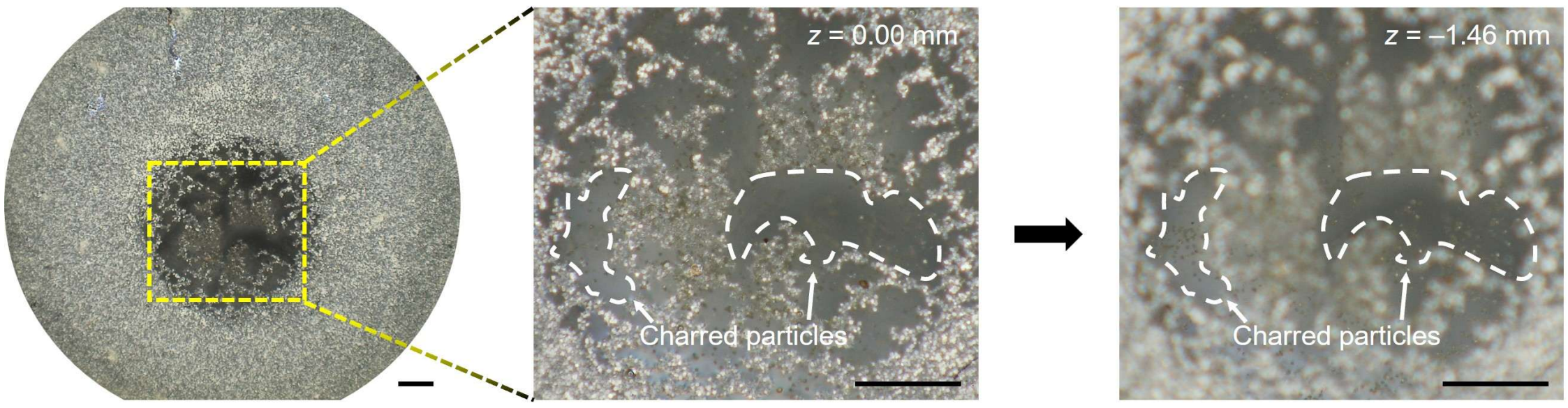

Fig. S3. The top of a droplet after laser irradiation, with an unjammed area amplified and imaged at different focal planes, revealing interior particles with reduced size and increased optical density (i.e., darkening). Scale bars, 1 mm.

Section S4: Viscosity dependence of scaling exponents and validation of the unjamming kinetics Model

This section presents a systematic investigation of centripetal surface flow and unjamming front dynamics across four glycerol–water solutions of varying viscosity, to validate the generality of the unjamming kinetics framework proposed in the main text. All experiments were conducted under the same standard configuration as the reference system (300-μL sessile droplets capped by a critically jammed lycopodium particle layer, 8 W laser irradiation), with only the solution volume fraction and corresponding viscosity varied.

For each viscosity condition, we extract two power-law exponents: the spatial exponent n describing the radial decay of surface velocity ($u \propto x^{-n}$) from particle-tracking velocimetry, and the temporal exponent α describing the subdiffusive expansion of the unjamming front ($d \propto t^{\alpha}$) from front-tracking measurements. The radial coordinate and flow velocity in our unjamming kinetics model are formally defined along the curved droplet surface (i.e., arc length from the apex and tangential surface speed). In experiments, both the front position d(t) and the radial surface flow velocity u(x) are measured from top-view imaging, corresponding to the horizontal projection radius and the in-plane radial speed, respectively. Within our entire observation window, the front propagates within the near-apex region, where the projected radial distance scales nearly linearly with the actual arc length along the curved interface. Notably, our droplets are formed via coalescence on a pinned three-phase contact line, which results in a flatter apex region than a perfect spherical droplet, further improving the accuracy of this linear approximation. Since a linear

scaling transformation only modifies the prefactor of a power-law relation and never changes the exponent, the curved-interface projection has no measurable influence on either the fitted power-law exponent of $d(t)$ or the spatial exponent n of the flow field.

With the measurement methodology clarified, we now examine whether the experimentally determined exponents satisfy the theoretical relation predicted by the unjamming kinetics model. Per the unjamming kinetics model, the two exponents α and n are linked by the intrinsic relation $\alpha = 1/(n + 1)$. Table S1 summarizes the experimentally measured exponents and the corresponding theoretical predictions for all tested viscosities.

Table S1. Measured and theoretical scaling exponents across all glycerol–water compositions. All data were obtained at 25 °C.

Glycerol volume fraction	Viscosity (mPa·s)	Spatial exponent n	Measured temporal exponent α	Theoretical prediction $1/(n+1)$
10%	1.19	0.94	0.47	0.52
40%	3.52	1.35	0.39	0.43
70%	22.4	1.76	0.34	0.36
80%	78.0	1.90	0.34	0.34

As solution viscosity increases, the spatial exponent n rises monotonically, and the deviation between the measured temporal exponent α and the theoretical prediction $1/(n+1)$ narrows progressively. This trend is physically consistent with the unjamming kinetics picture: at higher viscosity, the characteristic size of collectively unjamming particle clusters decreases, bringing the system closer to the ideal single-particle unlocking assumption underlying the model. The 70 vol % glycerol–water reference system used in the main text already operates near this ideal single-particle unjamming regime, which accounts for the close agreement between its experimental value (0.34) and theoretical prediction (0.36).

While Table S1 compiles the numerically extracted scaling exponents for quantitative comparison, the raw experimental profiles, including spatial velocity distributions and temporal front propagation curves, along with their corresponding power-law fits are presented in Fig. S4 for direct visual verification. The consistent trend across all tested viscosities confirms that the coupled spatiotemporal scaling, mediated by interfacial unjamming kinetics, is a general feature of the system rather than an artifact of one specific viscosity condition.

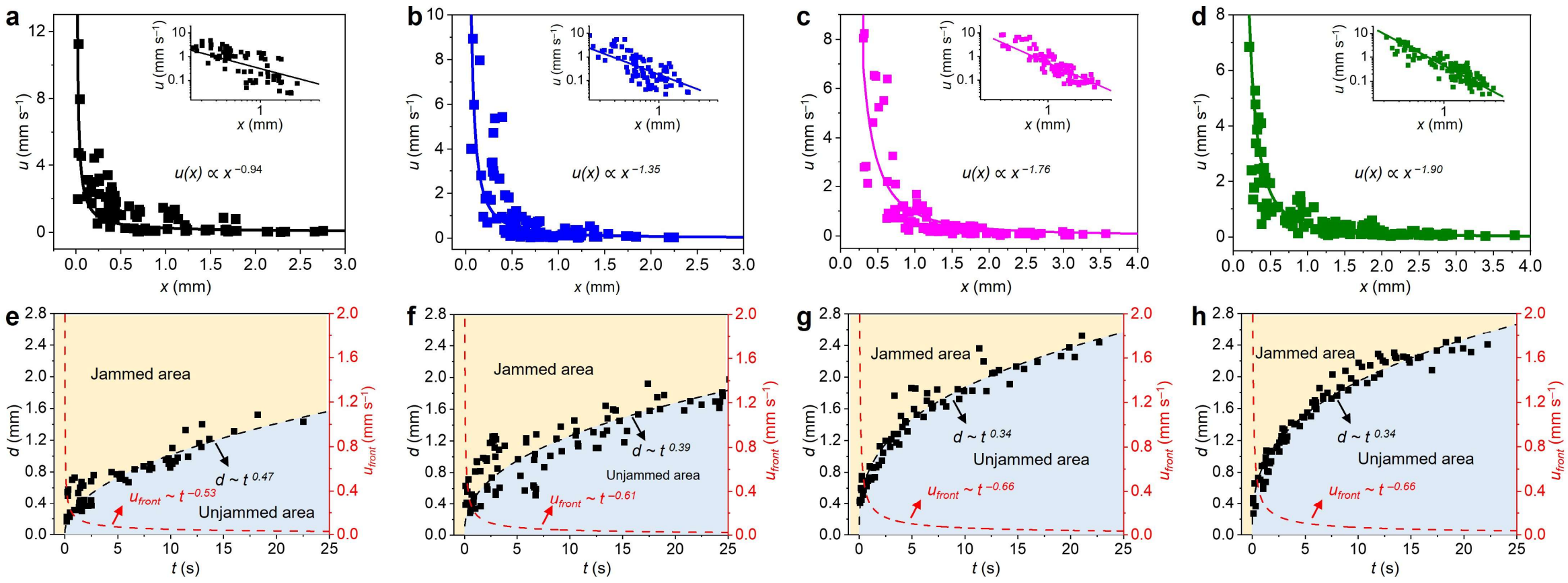

Fig. S4. Spatial velocity distributions (**a–d**) and temporal front propagation curves (**e–h**). Panels (**a–d**) present double-logarithmic plots revealing power-law scaling behavior (insets), while panels (**e–h**) show the corresponding front velocity profiles derived by numerical differentiation of the propagation curves. The glycerol–water solutions have glycerol volume fractions of 10 vol% (**a, e**), 40 vol% (**b, f**), 70 vol% (**c, g**), and 80 vol% (**d, h**).

Section S5. Measurement of the droplet side profile and curvature

Extracting and smoothing the side profiles of particle-capped droplets is essential for analyzing their morphological evolution during laser irradiation. To achieve this, we employed a method combining digital image processing and function fitting to generate smooth droplet contours. The procedure is as follows (Fig. S5):

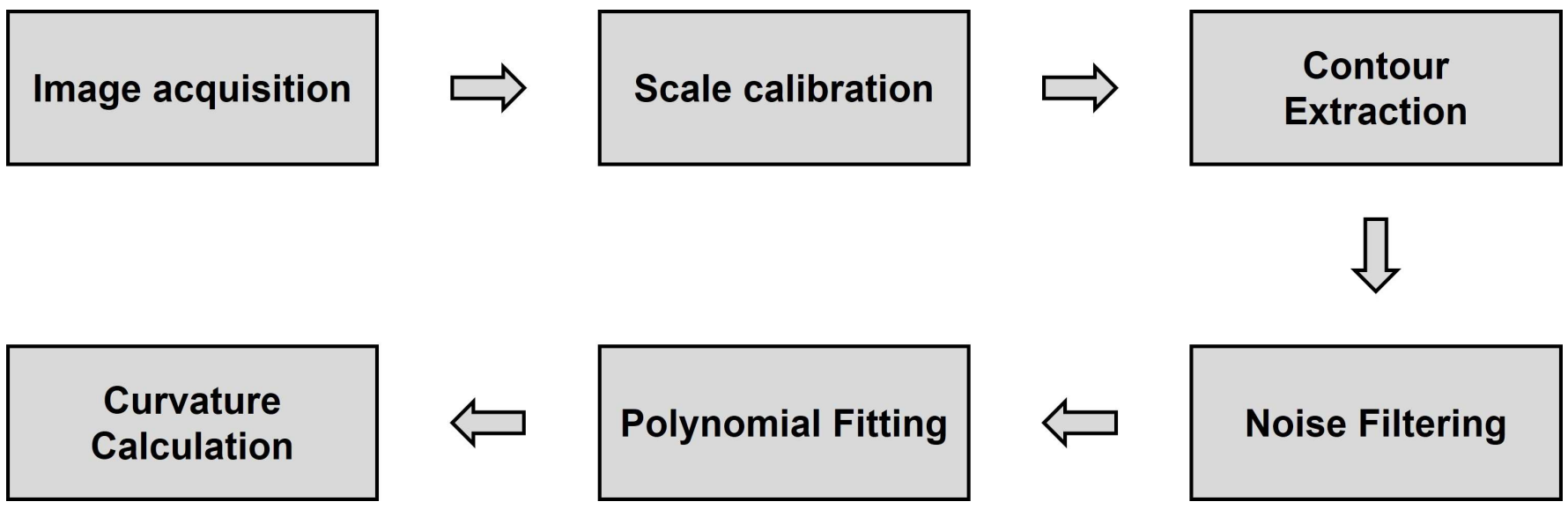

Fig. S5. Flowchart of droplet profile extraction and curvature measurement using digital image processing and function fitting.

First, after acquiring the side-view images of the droplet, a built-in dimensional reference object (the contact diameter of the droplet and the substrate) is used to establish the conversion relationship between pixel dimensions and actual physical scales. Subsequently, the droplet region is segmented from the background based on color difference. In the RGB color space, a threshold greater than 0.5 in the R-channel effectively separates the droplet area from the background. The

Canny edge detection algorithm (Gaussian filter coefficient σ = 1.5, double thresholds low=0.1/high=0.3) is applied to preliminarily identify the droplet boundary. Due to the presence of surface particles, the raw contour contains numerous local protrusions caused by particles, constituting high-frequency noise. To address this, a morphological closing operation (with a 2×2 pixel circular kernel) is used to initially smooth the rough contour. The smoothed pixel coordinates are then stored in a matrix for subsequent calculations.

To obtain a continuous, smooth geometric contour and facilitate subsequent curvature calculations, this study employs a polynomial function to fit the discrete points in the contour matrix. A coordinate system is established with the origin at the left triple contact point in the droplet's side view and the positive y-axis direction vertically upward. The contour fitting function is expressed as:

$$z(x) = \sum_{k=0}^{k} a_k x^k \tag{4.1}$$

Here, a_k represents the fitting coefficients. This study employs an 8th-order polynomial as the fitting function to approximate the droplet profile, i.e., $k = 8$. The choice of an 8th-order polynomial was made because analysis of the root mean square error (R^2) of the fitted curves revealed that when the highest order of the fitting polynomial is greater than or equal to 8, the R^2 value can consistently fall below 0.1 (with the maximum error being 1 and the minimum being 0), where:

$$R^2 = \frac{1}{n}\sqrt{\sum_{i=1}^{n} [z(x_i) - z_i]^2} \tag{4.2}$$

After obtaining the fitting function for the droplet profile, the function is substituted into the curvature equation, allowing the curvature function at different positions of the droplet to be derived. The MATLAB code for the droplet profile extraction and curvature calculation is as follows：

```
clear all;
clc;

mov = VideoReader('1.avi');
num = mov.NumFrames;
i = 0;

while 50*i+1 <= num
    %% Image acquisition and dimensional reference
    img = read(mov, 50*i+1);

    % Establish conversion relationship between pixel dimensions and physical scales
    % using built-in dimensional reference object (contact diameter)
    img_gray = img(600:946, 220:1120, :);

    %% Droplet segmentation based on color difference in RGB color space
    % Threshold greater than 0.5 in R-channel effectively separates droplet area
    img_bw = im2bw(img_gray, 0.45); % Adjusted threshold for better separation

    %% Canny edge detection algorithm
    % Gaussian filter coefficient σ = 1.5, double thresholds low=0.1/high=0.3
    img_edges = edge(img_bw, 'Canny', [0.1 0.3], 1.5);

    %% Morphological operations to smooth rough contour
    % Morphological closing operation with 2×2 pixel circular kernel
```

```
se = strel('disk', 2); % 2×2 pixel circular kernel
img_smoothed = imclose(img_edges, se);

%% Store smoothed pixel coordinates in matrix
[row, col] = find(img_smoothed);

%% Coordinate system establishment
% Origin at left triple contact point, positive y-axis vertically upward
pixel_to_mm = 15/1015; % Conversion factor from pixel to mm
x_coords = col * pixel_to_mm;
y_coords = (346 - row) * pixel_to_mm; % Flip y-axis to make upward positive

%% Polynomial function fitting for continuous smooth geometric contour
% 8th-order polynomial fitting function: y(x) = Σ(a_k * x^k) where k = 8
order = 8;

% Sort coordinates by x for proper polynomial fitting y(x)
[x_sorted, sort_idx] = sort(x_coords);
y_sorted = y_coords(sort_idx);

% Remove duplicate x values for stable polynomial fitting
[x_unique, unique_idx] = unique(x_sorted);
y_unique = y_sorted(unique_idx);

% 8th-order polynomial fitting
p = polyfit(x_unique, y_unique, order);

% Generate fitted curve
x_fit = linspace(min(x_unique), max(x_unique), 1000);
y_fit = polyval(p, x_fit);

%% Calculate root mean square error (R²) of fitted curves
y_actual = interp1(x_unique, y_unique, x_fit, 'linear', 'extrap');
residuals = y_actual - y_fit;
RMSE = sqrt(mean(residuals.^2));
R_squared = 1 - sum(residuals.^2) / sum((y_actual - mean(y_actual)).^2);

%% Substitute fitting function into curvature equation
% First derivative
dp = polyder(p);
dy_dx = polyval(dp, x_fit);

% Second derivative
d2p = polyder(dp);
d2y_dx2 = polyval(d2p, x_fit);

% Curvature function at different positions of the droplet
numerator = abs(d2y_dx2);
denominator = (1 + dy_dx.^2).^(3/2);
kappa = numerator ./ denominator;

%% Data output and visualization
% Save fitted profile coordinates
writematrix([x_fit', y_fit'], [num2str(i) ,'.xlsx'], 'Sheet', 1);

% Save curvature data
writematrix([x_fit', kappa'], [num2str(i) ,'.xlsx'], 'Sheet', 2);

% Visualization
subplot(2,1,1);
imshow(img_gray);
hold on;
plot(col, row, 'r.', 'MarkerSize', 1);
plot(x_fit/pixel_to_mm, 346 - y_fit/pixel_to_mm, 'g-', 'LineWidth', 2);
hold off;
title('Droplet Profile with Polynomial Fit');

subplot(2,1,2);
```

```
    plot(x_fit, kappa, 'b-', 'LineWidth', 2);
    title('Curvature Distribution along Droplet Profile');
    xlabel('x (mm)');
    ylabel('Curvature κ');

    % Store maximum values
    maxheight(i+1) = max(y_fit);
    maxqulv(i+1) = max(kappa);

    i = i + 1;
end

%% Final data organization
maxqulv = maxqulv';
maxheight = maxheight';
```

Section S6: Derivation of the balance condition for a sessile droplet

For a sessile droplet with a quiescent surface, force balance on a surface fluid element (unit width) dictates that the net pressure difference across the element balances the tangential gravitational component: $\frac{\partial P_i}{\partial s} = -\rho g \sin\theta$, where P_i is the internal liquid pressure, s is the arc length coordinate, ρ is density, g is gravitational acceleration, and θ is the acute angle between the surface tangent and horizontal. Given that height z decreases from apex to equatorial plane, $\frac{\partial z}{\partial s} = -\sin\theta$. Substituting $P_i = P_0 + \Delta p$ (where P_0 is atmospheric pressure, $\Delta p = \gamma\kappa$, γ is surface tension, and κ is curvature) yields:

$$\gamma \frac{\partial \kappa}{\partial s} = \rho g \frac{\partial z}{\partial s} \tag{6.1}$$

This equilibrium condition highlights the essential role of the curvature gradient in sustaining system stability.

Section S7. Energy-balance model for laser-induced apex-height decay evolution

We define η as the net effective energy-coupling efficiency, i.e., the fraction of incident laser power remaining for liquid vaporization (the latent heat of phase change) after optical absorption by the particle layer and subsequent deduction of all non-evaporative heat losses (conduction, convection, radiation, and particle pyrolysis). The net power available for liquid vaporization is therefore:

$$Q_{\mathrm{evap}} = \eta P \tag{7.1}$$

where *P* is the incident laser power. Denoting the latent heat of vaporization by *L*, the mass loss rate of the droplet satisfies:

$$\frac{dM}{dt} = -\frac{Q_{\mathrm{evap}}}{L} = -\frac{\eta P}{L} \tag{7.2}$$

With $M = \rho V$, where ρ is the liquid density and *V* the droplet volume, the volume change rate follows as:

$$\rho\frac{dV}{dt} = -\frac{\eta P}{L} \quad (7.3)$$

For our droplet with a pinned contact line (constant contact radius *R*), we approximate the droplet shape as a half-ellipsoid, for which $V = 2\pi R^2 h/3$. Substituting this into Eq. (7.3) and solving for *dh*/*dt* yields

$$\frac{dh}{dt} = -\frac{3\eta P}{2\pi\rho L R^2} \quad (7.4)$$

This result predicts that the apex height decreases at a constant rate for a given incident power, i.e., $h/h_0 = 1 - kt$ with $k = (3\eta P)/(2\pi\rho L R^2 h_0)$, and that the apex-height decay rate increases with laser power. Both features are qualitatively consistent with our experimental observations.

Section S8. Lubrication approximation and quantitative stress breakdown

8.1 Governing equations and equivalent surface tension definition

The lubrication approximation is applied to the flattened near-apex region of the droplet, justified by the small interface slope and low Reynolds number ($\mathrm{Re} \approx 0.14$) of the creeping flow. The radial momentum equation reduces to:

$$\frac{\partial p}{\partial x} = \mu\frac{\partial^2 u}{\partial z^2}$$

where x is the horizontal radial coordinate, z is the vertical coordinate normal to the substrate, μ is the dynamic viscosity, p is the liquid pressure, and u is the radial flow velocity. Integration is performed with two boundary conditions:

- No-slip condition at the substrate ($z = 0$): $u = 0$
- Tangential stress balance at the free surface ($z = h$): $\mu\partial u/\partial z = \partial\gamma/\partial x$, where γ is surface tension and $\partial\gamma/\partial x$ accounts for Marangoni stresses.

The resulting surface velocity is:

$$u_s = -\frac{h^2}{2\mu}\frac{\partial p}{\partial x} + \frac{h}{\mu}\frac{\partial\gamma}{\partial x}$$

To enable direct comparison between pressure-driven and capillary-driven mechanisms, we define an equivalent surface tension difference Σ such that $u_s \equiv (h/\mu)\Sigma$. For the pressure-driven contribution:

$$\Sigma_p = -\frac{h}{2}\Delta p$$

where Δp is the total pressure difference across the radial interval of interest. The total pressure difference combines Laplace pressure and hydrostatic pressure contributions:

$\Delta p = \gamma\Delta\kappa + \rho g\Delta z$

where $\Delta\kappa$ is the curvature difference across the interval, ρ is liquid density, g is gravitational acceleration, and Δz is the interface height difference across the interval.

8.2 Pressure-driven stress calculation

All calculations are performed for the 70 vol % glycerol–water model system at $t = 14\text{s}$, over the radial range 0–2.7 mm corresponding to the mobile unjammed zone. Input parameters and experimentally measured values are summarized below:

- Local liquid film height at the reference position: $h = 2.67\ \text{mm}$
- Surface tension: $\gamma = 0.065\ \text{N/m}$
- Liquid density: $\rho = 1180\ \text{kg/m}^3$
- Gravitational acceleration: $g = 9.8\ \text{m/s}^2$
- Measured curvature difference across 0–2.7 mm: $\Delta\kappa = 50.1\ \text{m}^{-1}$
- Measured interface height difference across 0–2.7 mm: $\Delta z = -0.08747\ \text{mm}$

Substituting these values yields:

- Laplace pressure difference: $\gamma\Delta\kappa = 3.2565\ \text{Pa}$
- Hydrostatic pressure difference: $\rho g\Delta z = -1.0115\ \text{Pa}$
- Total pressure difference: $\Delta p = 2.245\ \text{Pa}$

The corresponding equivalent surface tension differences are:

- Laplace contribution: $\Sigma_{\text{Lap}} = -\frac{h}{2}\gamma\Delta\kappa \approx -4.35\ \text{mN/m}$ (centripetal, negative by convention)
- Hydrostatic contribution: $\Sigma_{\text{hydro}} = -\frac{h}{2}\rho g\Delta z \approx +1.35\ \text{mN/m}$ (centrifugal, positive by convention)
- Net pressure-driven stress: $\Sigma_p = -3\ \text{mN/m}$ (net centripetal)

8.3 Marangoni stress calculation

The total Marangoni equivalent surface tension difference equals the total surface tension change across the 0–2.7 mm radial interval, comprising thermal and solutal components:

$$\Sigma_{\text{Ma}} = \Delta\gamma_T + \Delta\gamma_C$$

8.3.1 Thermal marangoni component

The radial temperature gradient was characterized experimentally using a fine dual-channel thermocouple inserted just beneath the liquid surface. A schematic of the temperature measurement configuration is provided in Fig. S8, illustrating the thermocouple placement at two radial positions: the droplet center directly below the laser irradiation zone, and the reference position at 2.7 mm from the center. The measured temperature difference across this 0–2.7 mm calculation interval is $\Delta T \approx 22$ K. Using a surface tension temperature coefficient of $\partial\gamma/\partial T = -0.08\ \text{mN/(m·K)}$ for 70 vol % glycerol–water solution, the thermal Marangoni contribution is:

$$\Delta\gamma_T = \left|\frac{\partial\gamma}{\partial T}\right|\Delta T = 1.76\ \text{mN/m}$$

directed centrifugally (positive sign).

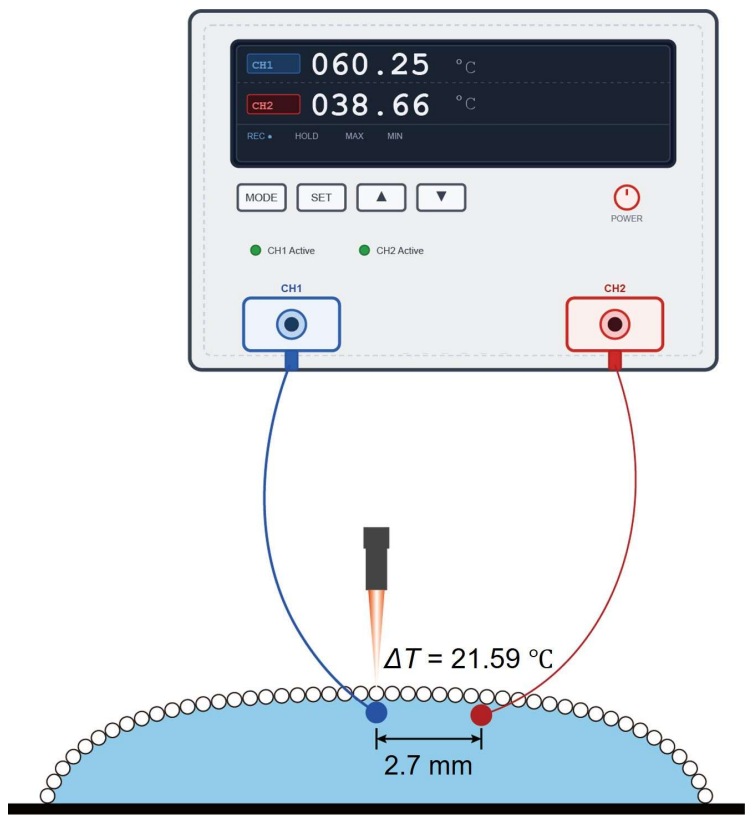

Fig. S8. Schematic illustration of the experimental setup for temperature measurement, showing simultaneous acquisition of temperature data at two spatially distinct positions.

8.3.2 Solutal Marangoni Component

The solutal Marangoni contribution is estimated conservatively, assuming a glycerol mass fraction difference of $\Delta C \approx 0.05$ across the 2.7 mm interval due to preferential water evaporation at the heated apex. Using a surface tension concentration coefficient of $\partial\gamma/\partial C \approx -9.4\ \mathrm{mN/m}$, where *C* is the glycerol mass fraction, the solutal Marangoni contribution is:

$$\Delta\gamma_C = \left|\frac{\partial\gamma}{\partial C}\right| \Delta C \approx 0.47\ \mathrm{mN/m}$$

also directed centrifugally. The total Marangoni equivalent stress is therefore:

$$\Sigma_{\mathrm{Ma}} = 1.76 + 0.47 = +2.23\ \mathrm{mN/m}$$

8.4 Solutal Rayleigh convection estimation

The contribution of solutal Rayleigh convection is estimated via an order-of-magnitude analysis. The equivalent interfacial shear stress from buoyancy-driven convection scales as:

$$\tau_{\mathrm{SR}} \sim \Delta\rho g \delta$$

where $\Delta\rho \approx 14.8\mathrm{kg/m^3}$ is the density difference induced by concentration gradients, and $\delta \approx$ 100 μm is an order-of-magnitude characteristic solute boundary layer thickness estimated via the diffusion length scale $\delta \sim \sqrt{Dt}$ (*D*: glycerol–water diffusion coefficient, *t*: characteristic evaporation timescale). Converted to an equivalent surface tension difference over the 2.7 mm radial interval, this yields:

$$\Sigma_{\mathrm{SR}} \approx 0.04\ \mathrm{mN/m}$$

directed centripetally. This value is two orders of magnitude smaller than the net pressure-driven stress, confirming its minor auxiliary role.

Movie S1. Surface flows on initially jammed and low-coverage droplets.

This is a comparative video of laser-induced surface flows. Under laser irradiation, the jammed

droplet undergoes continuous unjamming accompanied by centripetal particle migration, a flow direction opposite to classical Marangoni flow; in contrast, the unjammed droplet exhibits outward particle motion consistent with classical Marangoni flow.

Movie S2. Outward flow on an initially near-jamming droplet under a high laser power.

This video shows phenomena under near-jamming particle coverage and elevated laser power (28.0 W). Particles in the central region are rapidly dispersed outward within 1 s by strong Marangoni stresses, then accumulate and jam at the periphery of the depleted zone, demonstrating that this high laser power does not substitute for a pre-existing jammed layer.

Movie S3. Centripetal flow on an initially jammed pure water droplet.

A 300-μL pure water droplet exhibits the same centripetal migration and outward-expanding unjamming front observed in glycerol–water mixtures. Intermittent bubbles near the laser spot cause transient outward deflection of nearby particles, but the core centripetal flow pattern remains unambiguous. This result excludes solutal Rayleigh convection as the necessary driver.

Movie S4. Centripetal flow on initially jammed glycerol–water microdroplets.

This video shows two 3.5-μL microdroplets containing 80 vol% glycerol–water solution, displayed simultaneously in side view (left) and oblique view (right). Both microdroplets exhibit fast centripetal flow, consistent with the Laplace pressure mechanism.

SI References

(1) X. Li, Y. Wang, R. Wang, S. Wang, D. Zang, X. Geng, A dip-decoating process for producing transparent bi-superhydrophobic and wrinkled water surfaces. *Adv. Mater. Interfaces* 5, 1800356 (2018).
